# Decrease of excessive daytime sleepiness after shunt treatment for normal pressure hydrocephalus

**DOI:** 10.1111/jsr.14333

**Published:** 2024-09-14

**Authors:** Simon Lidén, Anna Lindam, Dan Farahmand, Anne‐Marie Landtblom, Katarina Laurell

**Affiliations:** ^1^ Department of Biomedical and Clinical Sciences, Neurology Linköping University Linköping Sweden; ^2^ Department of Public Health and Clinical Medicine, Unit of Research, Education and Development – Östersund Umeå University Umeå Sweden; ^3^ Department of Clinical Neuroscience, Institute of Neuroscience and Physiology, Sahlgrenska Academy University of Gothenburg Gothenburg Sweden; ^4^ Department of Medical Sciences, Neurology Uppsala University Uppsala Sweden

**Keywords:** cerebrospinal fluid shunts, Epworth sleepiness scale

## Abstract

Sleepiness and apathy are often reported in patients with normal pressure hydrocephalus. However, research on outcomes after shunt surgery has mainly focused on the classical triad symptoms, that is, gait, cognition, and bladder dysfunction. This study aimed to describe the effects of shunt treatment on excessive daytime sleepiness and whether there was a relation to changes in ventricular volume. Pre‐ and postsurgical excessive daytime sleepiness was investigated using the Epworth sleepiness scale in a sample of 32 patients with normal pressure hydrocephalus who underwent shunt surgery. Data were gathered before surgery and at 1, 2, and 3 months after surgery and with different settings of the shunt. In the total sample, the Epworth sleepiness scale improved by a median of 1.5 points at 1 month after surgery, *p* = 0.026. The improvement was predominately found in the group (*n* = 6) with high presurgical daytime sleepiness (Epworth sleepiness scale >12) (median = 12 points, *p* = 0.035) compared with a median change of 0 points (*p* = 0.47) in the group with Epworth sleepiness scale ≤12 (*n* = 26). Between the postsurgical follow‐ups, no further change in the Epworth sleepiness scale score was observed. The Epworth sleepiness scale score did not correlate with clinical tests nor with ventricular volume. Daytime sleepiness seems to be another domain of normal pressure hydrocephalus symptomatology in addition to the classical triad that is responsive to treatment, at least when pronounced. The Epworth sleepiness scale is a quick test to administer and could be a valuable addition to pre‐surgical screening for treatable symptoms.

## 1 INTRODUCTION

Idiopathic normal pressure hydrocephalus (iNPH) is a progressive neurological condition characterised by impaired cerebrospinal fluid (CSF) dynamics resulting in enlarged ventricles and often leading to the classical triad of gait disturbances, urinary incontinence, and cognitive decline (Hakim & Adams, 1965). The only effective treatment for iNPH is CSF flow diversion through shunt surgery (Giordan et al., 2018). In the clinical setting patients and caregivers often complain of tiredness, a symptom which can have many causes. Related symptoms such as apathy and depressive symptoms are reported to be frequent in patients with iNPH (Allali et al., 2018; Kito et al., 2009) and to improve with surgical shunt treatment (Israelsson et al., 2020; Peterson et al., 2016). Further, impaired wakefulness as observed during clinical visits improved following surgery and the improvement correlated to regional cerebral blood flow (Tullberg et al., 2004). Another study found a decrease in sleep need from 9 to 8 h per night following shunt surgery (Agerskov et al., 2018). A disruption of the glymphatic system (Iliff et al., 2013) has been seen in both patients with iNPH (Ringstad et al., 2017) as well as in individuals with impaired sleep (Eide et al., 2022), an improvement in the functioning of the glymphatic system following shunt surgery could be the cause of the improvement in wakefulness and the reduced need for sleep described previously (Israelsson et al., 2020; Peterson et al., 2016; Tullberg et al., 2004).

The Epworth sleepiness scale (ESS) (Johns, 1991) is a self‐rated scale used to evaluate daytime sleepiness, mostly used in sleep disorders such as narcolepsy and sleep apnea. The scale ranges from 0 to 24, with a higher score corresponding to a higher daytime sleepiness and an ESS score >10 has been suggested as a cut‐off for pathological values (Johns, 2000). Obstructive sleep apnea syndrome has been reported to be frequent in patients with iNPH, one study found a prevalence of 90% (*n* = 31) in iNPH patients seen at a memory clinic (Roman et al., 2018). In the same study, the mean ESS score was 11.4, although they did not examine whether there was improvement following surgery. Another study found a prevalence of sleep‐disordered breathing in iNPH of 96%, but a mean ESS score of only 5.9 and no difference between treated (*n* = 14) and untreated (*n* = 8) patients (Riedel et al., 2022). In investigations into the effects of shunt surgery on sleep disordered breathing, no improvement but a shorter overall sleep time has been seen (*n* = 17) (Kristensen et al., 1998).

Inspired by our clinical experience of complaints of sleepiness from patients with iNPH and their caregivers, the aim of this study was to investigate the presurgical excessive daytime sleepiness in patients with iNPH using ESS and whether there was an effect of shunt surgery on excessive daytime sleepiness. Secondary outcomes were correlations between ESS score and other iNPH symptoms of memory impairment, gait disturbance, and incontinence as well ventricular volume.

## 2 METHODS

This was a prospective study enrolling participants consecutively at the regional hospital in Östersund, Sweden between the period of 2017–2021. It was a part of a larger study on the effects of randomised shunt setting where the primary outcome of ventricular volume (VV) has been published previously (Liden et al., 2023). The inclusion criteria were a diagnosis of iNPH and planned shunt surgery. Exclusion criteria were a Mini Mental State Examination (MMSE) score of <16, contraindications to MRI, anticoagulant or antiplatelet medication beyond acetylsalicylic acid. In total 36 participants were enrolled in the study, 25 men and 11 women with a median (Md) age 76 (range 65–85) years, of which 32 had presurgical ESS data. See Table 1 for participant demographics.

**TABLE 1:** Participant characteristics.

	Total sample *n* = 32 Md (IQR)	High initial ESS *n* = 6 Md (IQR)	Low initial ESS *n* = 26 Md (IQR)
Age (yrs)	77 (72, 79)	75 (71, 79)	76.5 (72, 80)
BMI	26.2 (23.4, 30.6)	23.0 (21.4, 25.3)	27.6 (25.5, 31.3)
Volume (mL)	129 (116, 156)	146 (123, 155)	127 (115, 153)
TUG time (s)	16.3 (13.2, 20.3)	18.4 (16, 26.5)	16 (12.7, 20.1)
TUG steps	20 (16, 26)	24 (19, 40)	20 (16, 26)
10 W time (s)	13.1 (10.4, 17.7)	16.7 (11.4, 22.4)	12.6 (10.6, 17.3)
10 W steps	21 (18, 28)	24 (18, 47)	20 (18, 28)
MMSE	27 (25, 29)	28 (23, 28)	27 (25, 29)
ESS	5.5 (3, 10.5)	16 (15, 17.8)	4.5 (3, 7)

*Note*: Data presented as median (interquartile range). High initial ESS group with presurgical ESS score >12, while the low initial group had an ESS score of ≤12.

Abbreviations: 10 W, 10 m walking test; BMI, body mass index; ESS, Epworth sleepiness scale; MMSE, mini mental state examination; TUG, time up and go.

Clinical information including the tests; ESS, MMSE, Timed Up and Go (TUG), 10‐metre walking test (10 W), and incontinence rating was collected before surgery and at 1, 2, and 3 months afterwards. At the same visits the participants underwent MRI imaging with ventricular volume (VV) measurements. At surgery, all participants received a Strata® adjustable shunt at performance level (PL) 1.5. After each follow‐up, the PL of the shunt was changed according to randomisation arm, to which participants were assigned at the first follow‐up. These randomisation arms were blinded to both participants and clinical evaluator and consisted of a series of PL settings: either 2.5–1.0 or 1.0–2.5. Additionally, a final assessment of VV after 24 h at PL 0.5 was performed without ESS rating due to the difficulties for the participants to assess excessive daytime sleepiness for only the last 24 h. Automatic measurements of intracranial volume were provided by the SyMRI® post‐processing software and used to calculate the ratio of ventricular volume to intracranial volume (VV/ICV ratio). A more detailed description of the protocol and ventricular measurements is available in a previous publication (Liden et al., 2023).

The ESS consists of eight Likert items with possible values of 0–3 describing everyday scenarios and asking the participant to rate his or her risk of falling asleep in each scenario. The total sum of the items is then used as the scale score giving a possible range of 0 (no risk of falling asleep) and 24 (high risk of falling asleep in all scenarios). An investigator asked the questions and filled in the questionnaire and the participants were aided in answering the questions by relatives or caretakers when needed. A cut‐off for pathological ESS of >10 has previously been suggested, although it is based on data from studies on narcolepsy and younger populations (Johns, 2000). For men in the age range 65+ years, published data on a sample from the general population described a mean ESS score of 6.1 with standard deviation 3.6, and where 12.7% had an ESS of >10 (Spira et al., 2012). For women in the age range 70+ years, a median score of 5 with interquartile range 3–8 was described while 10.8% had an ESS of >10 (Beaudreau et al., 2012). Based on this, a cut‐off of >12 for the classification as pathological was chosen for this study to better represent ESS values in the older population.

Improvement after surgery was defined as at least one of the following (a–c) as has been done previously (Kockum et al., 2020; Liden et al., 2022; Liden et al., 2023; Virhammar et al., 2014):

- A 20% reduction in the time or the number of steps in at least one of the two motor function tests (TUG, and 10 m walk).
- Four points increase in the Mini‐Mental State Examination.
- One level increase in the continence scale and two points increase in the Mini‐Mental State Examination.

### 2.1 Statistics

Significance was set at *α* = 0.05. The sample size was determined by power calculation for clinical walking tests for the larger study, not presented here. Due to that, the ESS did not follow the normal distribution, non‐parametric methods of analysis were used as appropriate: Mann–Whitney U‐test, Wilcoxon signed‐rank, Spearman correlation. An aligned rank transformed linear mixed model assessed with ANOVA was used as an omnibus test (Wobbrock et al., 2011). Statistical analysis was performed using R version 4.2.2 (R Foundation for Statistical Computing, Vienna, Austria).

## 3 RESULTS

Of the 36 participants, 72% had improved clinically at the first follow up 1 month after surgery. Before surgery, the median ESS score was 5.5 with a range of 1–23 and interquartile range (IQR) of 3–10.5. The omnibus test was significant at *p* = 0.048. At the first follow up 1 month after surgery the median ESS score was 4 with a range of 1–13 and an IQR of 3–7, resulting in a median improvement of 1.5 points, *p* = 0.026. This improvement remained for the duration of the study regardless of shunt setting or timepoint as summarised in Table 2. However, only 18 finished the full protocol. There were no significant differences in ESS between any two postsurgical timepoints or shunt PL, *p* > 0.24 for all pairwise tests.

**TABLE 2:** Post surgical ESS score change by timepoint or shunt setting.

Follow up scenario	One month^a^	Two months	Three months	PL 2.5	PL 1.0
Md ESS change (IQR)	−1.5 (−4.25, 1)	−2 (−7.5, 0.5)	−2 (−4, 0.625)	−2 (−5, 0)	−2 (−8.25, 0.875)
*p*‐value	0.026	0.014	0.043	0.027	0.025
*n*	32	23	16	21	18
*n* ESS > 12	1	0	0	0	0

*Note*: *p*‐value for difference in ESS score between the follow‐up and presurgical values.

^a^ Shunt setting at 1 month was PL 1.5 for all participants.

**FIGURE 1:**
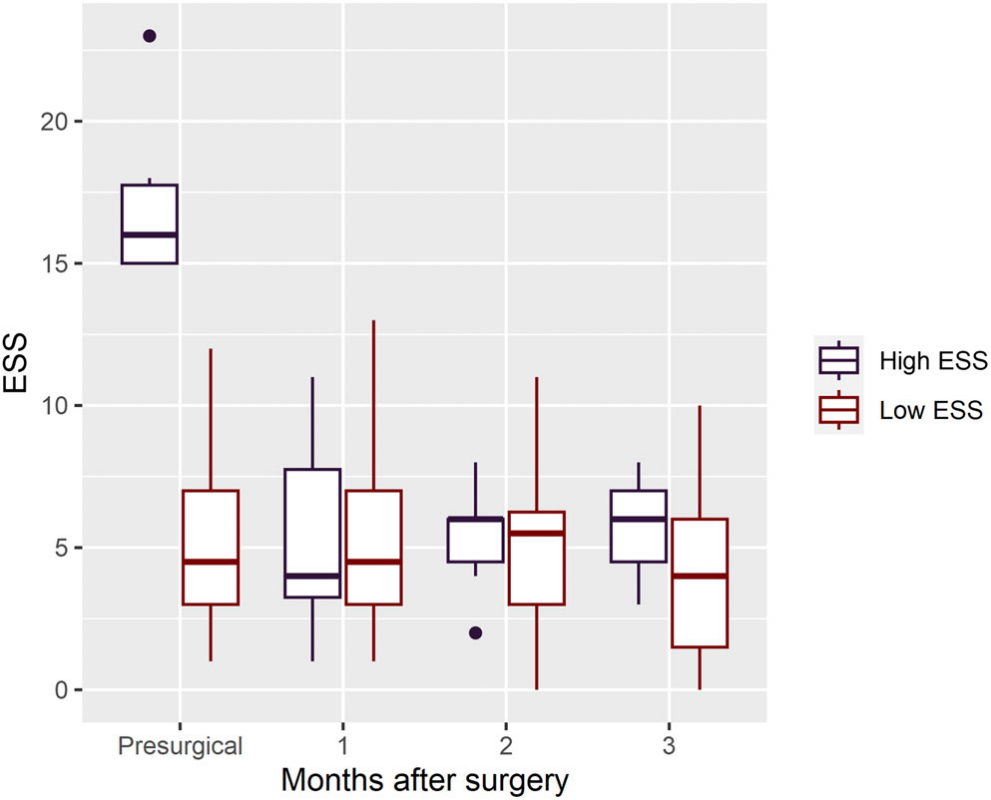
Box plot of Epworth sleepiness scale (ESS) score by high or low initial ESS. Cut‐off ESS >12.

A post hoc analysis dividing the participants by presurgical ESS with a cut‐off of >12 revealed that the group with a high initial ESS score carried the effect. The median change after surgery in that group (*n* = 6) was −12 (*p* = 0.035), whereas the median change in the low ESS group (*n* = 26) was 0 (*p* = 0.47), visualised in Figure 1. There were no significant differences between the groups in presurgical MMT, iNPH‐scale score, TUG time, age, nor VV. In the high ESS group, one participant had a preexisting diagnosis of obstructive sleep apnea and an unchanged treatment for the condition during the study period. No other participant in that group received an obstructive sleep apnea diagnosis, nor treatment for the condition during the follow‐up period. The body mass index (BMI) of participants was also lower in the high ESS group. There were no clinical signs of other neurodegenerative disease, as an alternative explanation of EDS, in the presurgical neurological evaluation nor during the follow‐up period of 2–3 years in the high ESS group.

Before surgery, there was a trend toward a correlation between the VV/ICV ratio and the ESS score, *p* = 0.061, Spearman's rho = 0.33. There was no correlation between the change in ESS score and the change in VV/ICV ratio after surgery, *p* = 0.42. There was no correlation between MMSE, TUG or 10 m walking time and the ESS in absolute values before surgery (*p* = 0.92, *p* = 0.39, *p* = 0.79) nor in change after surgery (*p* = 0.66, *p* = 0.88, *p* = 0.83).

## 4 DISCUSSION

In this study we investigated excessive daytime sleepiness, as measured by ESS, before and after shunt surgery for normal pressure hydrocephalus which, to our knowledge, has not previously been done. The main finding was an improvement of excessive daytime sleepiness following surgery. This result is in accord with previous reports on improved wakefulness (Tullberg et al., 2004), as well as reduced total sleep time (Agerskov et al., 2018; Kristensen et al., 1998) for patients with iNPH following surgery.

A subgroup of participants with a high ESS score (>12) improved markedly after surgery. After the first month, there was no further change in ESS regardless of the shunt setting or timepoint. This sustained improvement with no trend of returning to presurgical values during the follow‐up period is reassuring as a self‐reported rating scale such as ESS might be susceptible to placebo after surgery. The division of the material in a high and low ESS group is not unproblematic when subsequently assessing a change in the same score, as it is vulnerable to regression to the mean and an artificial decrease in the *p*‐value. Therefore, the subgroup analyses should only be viewed as an illustration of where the effect in the full sample originated rather than interpreted in isolation. The fact that only one participant in the ESS ≤12 group increased to ESS >12 during the follow‐up period indicates that the initially high ESS scores are signs of a stable presurgical daytime sleepiness rather than the results of random fluctuations of ESS.

In the timeframe of 1 month, the ESS did not change depending on the shunt setting. This could be either because any drainage through the shunt in the tested range of opening pressure is enough to alleviate the shunt‐responsive excessive daytime sleepiness, or that changes in excessive daytime sleepiness take longer to manifest. While our study was not designed to further assess these possibilities, the rapid improvement already by 1 month after surgery would suggest the former.

As excessive daytime sleepiness seems to improve following treatment for iNPH, the question remains as to the mechanism of action. One interesting possibility is an improvement in glymphatic functioning following shunt surgery. Previous investigations have seen a lower diffusion tensor image analysis along the perivascular space (DTI‐ALPS) index, a MRI‐based measurement of diffusion along white matter venous perivascular spaces, in shunt non‐responders when compared with responders as a possible marker for a more affected glymphatic system (Bae et al., 2021), as well as an increase in the DTI‐ALPS index following lumboperitoneal shunt surgery (Kikuta et al., 2022). However, the interpretation of the DTI‐ALPS index as a glymphatic marker is controversial (Ringstad, 2024) and it is possible that the observations are a consequence of other changes in the white matter in iNPH.

Previous work revealed a high score of Apathy Evaluation Scale in iNPH that also improved after surgery (Peterson et al., 2016). There is a possibility of ESS and Apathy Evaluation Scale evaluating similar aspects of NPH symptomatology and underlying pathological mechanisms. This should preferably be explored in future studies using both scales on the same sample of iNPH patients. However, one indication that the two scales do actually measure different symptoms is that we did not find a significant correlation between ESS and cognitive function assessed with MMSE which was found in the study with the Apathy Evaluation Scale (Peterson et al., 2016). Another study saw an improvement in wakefulness as observed by clinicians following shunt surgery for NPH (Tullberg, 2004). While we collected no such data in this study, there would likely be a strong correlation between observations of wakefulness and ESS score as they both track the patient's propensity to fall asleep.

Although objectively registered sleepiness is likely to have superior precision, a self‐reported instrument might be practical to use for screening of the symptom in a clinical setting. As far as we know, this is the first study on the effects of shunt surgery on daytime sleepiness in iNPH and no disease specific questionnaire is available. The existing questionnaires for sleep assessment are not adapted for a geriatric population (Frohnhofen et al., 2020). We chose ESS because it is easy to administer and can be completed by patients on their own or helped by relatives or caregivers as needed, with little cost of healthcare resources. As ESS seems to measure an aspect of NPH symptomatology amenable to treatment and does not correlate strongly with other symptoms, the inclusion of ESS at presurgical workup could be a valuable and cost‐efficient addition to clinical routine.

### 4.1 Strengths and limitations

There was a high improvement rate in clinical symptoms and in line with previous research (Giordan et al., 2018; Sundstrom et al., 2017) indicating a representative sample of patients with iNPH without significant comorbidity of other neurodegenerative disorders. The relatives and caretakers were allowed to help the participants in completing the ESS questionnaires, but variation in who accompanied the participants to the clinical evaluations and subsequently what aid was available to them at each follow‐up point, is a potential source of error. While we found no correlation between outcomes in the ESS and clinical tests, the limited sample size was not powered to rule out such an association. The ESS assessment was performed after the participants had been offered surgery, removing the risk for bias of having it included in the presurgical evaluation. As the participants were not selected by the ESS score, together with the lasting improvement in ESS scores, the risk of a meaningful regression to the mean is low for the full sample, although it remains for the subgroup analysis. As there was no control group there is a risk of a meaningful placebo component in the findings of the study as there are indications that placebo effects after surgery can endure over long periods of time (Wartolowska et al., 2016). The *p*‐values reported in the study were not corrected for multiple comparisons but the consistent, stable improvement at all follow‐up points support the validity of our findings.

## 5 CONCLUSION

Our results indicate that excessive daytime sleepiness might be an additional symptom to the classical triad in patients with iNPH. The ESS seems to be useful in the presurgical diagnostic workup of patients with iNPH. However, due to the exploratory nature of this study the results would need to be replicated and preferably validated with objective registration methods, before recommending the routine use of ESS to screen for treatable symptoms. Of interest in future studies on excessive daytime sleepiness in iNPH would be whether there are any correlations with apathy, impaired wakefulness, and obstructive sleep apnea.

## AUTHOR CONTRIBUTIONS

**Simon Lidén:** Investigation; funding acquisition; writing – original draft; writing – review and editing; formal analysis; project administration; data curation; methodology; visualization. **Anna Lindam:** Methodology; writing – review and editing; formal analysis. **Dan Farahmand:** Conceptualization; writing – review and editing; methodology; supervision. **Anne‐Marie Landtblom:** Conceptualization; writing – review and editing; supervision. **Katarina Laurell:** Conceptualization; investigation; funding acquisition; writing – review and editing; methodology; supervision; resources; project administration.

## FUNDING INFORMATION

The study has received funding from the following sources: Region Jämtland Härjedalen Research and Development Department; Uppsala University; Forskningsfonden för klinisk neurovetenskap vid Norrlands Universitetssjukhus; Jämtland läns cancer och omvårdnadsfond; Syskonen Perssons donationsfond; NEURO Sweden.

## CONFLICT OF INTEREST STATEMENT

None of the authors have any conflicts of interest to disclose.

## PATIENT CONSENT

All participants received written and verbal information and signed a consent form.

## TRIAL REGISTRATION

The study was registered at clinicaltrials.gov with identification number NCT04599153.

## Data Availability

The data that support the findings of this study are available on request from the corresponding author after appropriate ethical review board approval.
